# Draft Genome Sequence of Labrenzia sp. Strain EL143, a Coral-Associated Alphaproteobacterium with Versatile Symbiotic Living Capability and Strong Halogen Degradation Potential

**DOI:** 10.1128/genomeA.00132-18

**Published:** 2018-03-08

**Authors:** Gisele Nunes Rodrigues, Asunción Lago-Lestón, Rodrigo Costa, Tina Keller-Costa

**Affiliations:** a IBB–Institute for Bioengineering and Biosciences, Department of Bioengineering, Instituto Superior Técnico, Universidade de Lisboa, Lisbon, Portugal; b Centro de Investigación Científica y de Educación Superior de Ensenada (CICESE), Ensenada, Mexico

## Abstract

We report here the genome sequence of Labrenzia sp. EL143, an alphaproteobacterium isolated from the gorgonian coral Eunicella labiata that possesses various genes involved in halogen and aromatic compound degradation, as well as polyketide synthesis. The strain also maintains multiple genes that confer resistance to toxic compounds such as heavy metals and antibiotics.

## GENOME ANNOUNCEMENT

Labrenzia species are Gram-negative, motile, rod-shaped bacteria that require NaCl for growth (1). There are six recognized species, all isolated from marine habitats and often associated with host organisms such as dinoflagellates, halophytes, and oysters (2). So far, 18 Labrenzia genome sequences are available in public databases. Although the symbiotic association between Labrenzia species and corals has been reported (3–5), genome-wide assessments of coral-associated Labrenzia species have not been performed. Strain EL143 was isolated from the gorgonian coral Eunicella labiata, sampled at a depth of 18 m off the Atlantic coast of Faro Beach, Algarve, Portugal (36°58′47.2″N, 7°59′20.8″W) (5). In the laboratory, host-derived microbial cell suspensions were prepared as described elsewhere (6) and inoculated onto 1:2 diluted marine agar medium for 4 weeks at 18°C (5). Genomic DNA from a pure EL143 culture was extracted using the Wizard Genomic DNA purification kit (Promega) according to the manufacturer’s instructions and sequenced on an Illumina MiSeq platform, as previously described (7, 8). The sequence output totaled 720 Mb, consisting of 1,196,456 reads (2 × 301 bp), with a genome coverage of 99×. Sequence reads were assembled *de novo* into 34 contigs using the NGen DNA assembly software (DNAStar, Inc.). NCBI BLAST analyses suggest that 11 of the 34 contigs belong to plasmids. Gene prediction and annotation were performed with the Rapid Annotations using Subsystems Technology (RAST) prokaryotic genome annotation server, version 2.0 (9). The antiSMASH platform was used to identify biosynthetic gene clusters (10).

The genome is composed of 7,279,421 bp, with a calculated G+C content of 56.3%. It possesses 7,614 coding sequences, in addition to 49 RNAs. Labrenzia sp. EL143 shares 99.8% 16S rRNA gene homology with L. alba (formerly Stappia alba) 5OM9, isolated from Mediterranean oysters (1, 11), and 98.0% 16S rRNA gene homology with the type strain of the species, L. alba CECT 5094 (1, 11).

Labrenzia sp. EL143 carries many genes that support the symbiotic relationship with sessile hosts, including 71 genes involved in resistance to antibiotics and toxic compounds, 15 *Czc* (cobalt-zinc-cadmium resistance) genes presumably located on a plasmid, and various genes encoding multidrug resistance efflux pumps. Moreover, the genome possesses strong dehalogenation potential, comprising four haloacid dehalogenase encoding genes and one haloalkane dehalogenase (EC 3.8.1.5) encoding gene involved in the degradation of a wide range of aliphatic and aromatic halogens, haloalcohols, and haloacids. Since the oceans contain a variety of halogenated compounds, bacterial symbionts of filter-feeding marine organisms such as gorgonians presumably use dehalogenases for detoxification and nutrition (12). Strain EL143 may further degrade a large number of aromatic compounds, including polychlorinated biphenyls (via EC 1.13.11.39), benzoate (via EC 4.1.1.7), and catechol via the β-ketoadipate pathway (at least 20 genes), corroborating earlier results for L. alba strains 5OM6, 5OM9, and 5OM30 (11). Labrenzia sp. EL143 also harbors genes related to chitin utilization, including one endochitinase (EC 3.2.1.14) and three *N*-acetylglucosaminidases (EC 3.2.1.52). Moreover, type I (thiopeptide-transAT polyketide synthase [PKS]-nonribosomal peptide synthetase, 102 kb) and type III (31 kb) PKS gene clusters, in addition to a thiopeptide (27 kb) and a terpene (20 kb) biosynthetic cluster, are present.

### Accession number(s).

The genome sequence of Labrenzia sp. EL143 has been deposited in the European Nucleotide Archive under the accession numbers OGUZ01000001 to OGUZ01000034. The study ID is PRJEB24678, and the sample ID is ERS2166916.
